# Diversity of microbiota in Slovak summer ewes’ cheese “Bryndza”

**DOI:** 10.1515/biol-2021-0038

**Published:** 2021-03-23

**Authors:** Miroslava Kačániová, Margarita Terentjeva, Simona Kunová, Peter Haščík, Przemysław Łukasz Kowalczewski, Jana Štefániková

**Affiliations:** Department of Fruit Science, Viticulture and Enology, Faculty of Horticulture and Landscape Engineering, Slovak University of Agriculture, Tr. A. Hlinku 2, 94976 Nitra, Slovakia; Department of Bioenergetics, Food Analysis and Microbiology, Institute of Food Technology and Nutrition, University of Rzeszow, Cwiklinskiej 1, 35-601, Rzeszow, Poland; Institute of Food and Environmental Hygiene, Faculty of Veterinary Medicine, Latvia University of Life Sciences and Technologies, K. Helmaņaiela 8, LV-3004, Jelgava, Latvia; Department of Food Hygiene and Safety, Faculty of Biotechnology and Food Sciences, Slovak University of Agriculture, Tr. A. Hlinku 2, 94976 Nitra, Slovakia; Department of Technology and Quality of Animal Products, Faculty of Biotechnology and Food Sciences, Slovak University of Agriculture, Tr. A. Hlinku 2, 94976 Nitra, Slovakia; Department of Food Technology of Plant Origin, Poznań University of Life Sciences, 31 Wojska Polskiego St., 60-624 Poznań, Poland; AgroBioTech Research Centre, Slovak University of Agriculture, Tr. A. Hlinku 2, 94976 Nitra, Slovakia

**Keywords:** gram-positive and gram-negative bacteria, microscopic filamentous fungi, sheep farms, ewes milk cheese “Bryndza”, mass spectrometry, microbiota identification

## Abstract

“Bryndza” cheese is an important Slovak traditional regional product. New knowledge on the role of microorganisms involved the “Bryndza” ripening process may provide valuable data on its quality and safety. In our study, the “Bryndza” made from pasteurized ewes milk was studied towards total count of bacteria, coliforms bacteria, enterococci, lactic acid bacteria, and microscopic filamentous fungi. All those groups of microbiota were detected using classical microbiological methods and identified using mass spectrometry. A total of 3,758 isolates were identified with score higher than 2.00. Altogether, 13 families, 24 genus, and 44 species of microbiota were identified in Slovak cheese “Bryndza.” The most often isolated species were yeasts *Yarrowia lipolitica* and *Dipodascus geotrichum* and the lactic acid bacteria *Lactobacillus paracasei* subsp. *paracasei*.

## 1 Introduction

Sheep herding and ewes milk production are important sectors of Slovak agriculture. The well-known product of ewes milk is a Slovak cheese named “Bryndza” or “Oštiepok” [1,2]. “Bryndza” was granted the Protected Geographic Indication (PGI) as it is produced in a defined mountainous regions of Slovakia [3,4]. “Bryndza” is a type of natural white, mature, and spreadable cheese made from matured 100% sheep lump cheese or mixed with up to 50% of cow lump cheese. First, ewes milk is sweetened. This raw material is then allowed to dry on the mountain hut to form lump sheep’s cheese. It is then transferred to a bryndziarna (cheese “Bryndza” production), where it is sorted and washed with water and then left to mature at 20°C in a cheese bath. The rind is then removed from the cheese, the excess liquid (whey) is expelled, and the cheese is crushed. The pulp is salted and spread on rollers to form a “Bryndza” [5]. The manufacturing has been done according to the traditional method. “Bryndza” is produced from raw ewes milk at 29–31°C for 30 min with chymosin or chymosin-identical enzymes [6]. In 2019, the production of ewes milk in Slovakia reached 13,524 tons, which is the highest level since joining the European Union. The annual production of “Bryndza” in Slovakia is almost 4,000 tons, whereas Slovaks consume about 0.6 kg of “Bryndza” per capita per year. The characteristics of the produced “Bryndza” cheese may vary under the climatic conditions and may be influenced by the feeding meant. The botanical composition of the plants in the sheep diet during pasture may affect the quality parameters of the milk used for production of the “Bryndza” cheese. Carpathian Mountains in Slovakia thus represent a specific habitat for sheep milk production by affecting the quality of raw milk [7] and the differences in ambient temperatures influence the ripening microbiota at the early cheese production steps [8]. In Slovak “Bryndza,” *Hafnia alvei* and *Klebsiella oxytoca* were the most abundant Gram-negative bacteria, whereas *Lactococcus lactis* and *Lactobacillus paracasei* the most abundant Gram-positive bacteria. *Lactobacillus, Lactococcus*, and *Pediococcus* were the main representatives of the lactic acid bacteria [8]. The aim of this study was to characterize microbiological variability of “Bryndza” cheese produced in summer of 2016–2019 in various geographical areas in Slovakia.

## 2 Materials and methods

### 2.1 “Bryndza” cheese samples

Samples of 80 unpasteurized ewes’ cheese “Bryndza” were provided by eight producers representing eight Slovak farms (F1 to F8). Slovak “Bryndza” is produced in the same way throughout the defined area. The same breed of sheep grazes in the defined area – Native Wallachian sheep, Improved Wallachian sheep, Domestic Tsigai, and East Friesian on pastures with the same flora and climatic conditions, which results in the same quality of the basic raw material – ewes milk [4]. The samples were collected between the end of April and August in the years 2016 to 2019. All samples were transported to the laboratory at 4°C and were analyzed immediately after delivery. Each sample was analyzed for total count of bacteria, coliform bacteria, enterococci, lactic acid bacteria, microscopic fungi, and yeasts MFF (microscopic filamentous fungi). The serial dilutions of the milk products in 0.89% sterile saline were made for plating out the sample material.

### 2.2 Microbiological analysis

The determination of total count of bacteria, coliforms, enterococci, lactic acid bacteria and fungi, and yeasts has been previously published [9,10]. The colonies from total count of bacteria, coliforms bacteria, enterococci, lactic acid bacteria, MFF, and yeasts were selected for further confirmation with MALDI-TOF MS Biotyper. Selected colonies were subcultured overnight on TSA agar aerobically or anaerobically and were used for identification.

### 2.3 Identification of bacteria and yeasts with MALDI-TOF MS Biotyper

Microbial isolates for MALDI-TOF MS Biotyper analysis were prepared in accordance with extraction procedure provided by the manufacturer (Bruker Daltonics, Bremen, Germany). The detailed procedure was previously published [11].

### 2.4 Identification of microscopic filamentous fungi with MALDI-TOF MS Biotyper

The detailed procedure of identification of fungal isolates was previously published [12]. Identification was done by MALDI-TOF MS Biotypes (Bruker Daltonics, Bremen, Germany) with Flex Control 3.4 software and Biotyper Realtime Classification 3.1 with BC specific software (Bruker Daltonics, Germany).

### 2.5 Krona charts

Krona charts showing automatically the taxonomic identification and relative abundance of the most abundant bacteria or microscopic filamentous fungi.

### 2.6 Statistical analysis

For microbial counts, lactic acid bacteria count, coliform bacteria, and microscopic filamentous fungi counts, the means and standard deviations were calculated. Krone diagrams were used for visualization of the relatedness of the identified microbial isolates.

## 3 Results

The number of coliform bacteria ranged from 3.67 in F1 to 3.84 log cfu/g in F2 and enterococci from 2.29 in F6 to 2.64 log cfu/g in F4. Total count of bacteria were from 4.38 in F1 to 4.73 log cfu/g in F4. The higher lactic acid bacteria counts were found in F7 (3.62 log cfu/g) and microscopic filamentous fungi in F6 (2.61 log cfu/g) (Table 1).

**Table 1:** Microbial counts in sheep cheese “Bryndza” (average ± SD log cfu/g)

Farm	CB	E	TCB	LAB	MFF
F1	3.67 ± 0.15	2.46 ± 0.13	4.38 ± 0.15	3.25 ± 0.12	2.42 ± 0.13
F2	3.81 ± 0.19	2.42 ± 0.12	4.66 ± 0.11	3.37 ± 0.15	2.36 ± 0.12
F3	3.79 ± 0.20	2.27 ± 0.17	4.64 ± 0.17	3.42 ± 0.17	2.15 ± 0.11
F4	3.81 ± 0.19	2.64 ± 0.11	4.73 ± 0.12	3.43 ± 0.12	2.26 ± 0.12
F5	3.76 ± 0.12	2.36 ± 0.10	4.48 ± 0.11	3.37 ± 0.15	2.41 ± 0.15
F6	3.72 ± 0.12	2.29 ± 0.14	4.81 ± 0.12	3.26 ± 0.11	2.61 ± 0.12
F7	3.74 ± 0.11	2.32 ± 0.11	4.64 ± 0.15	3.62 ± 0.12	2.41 ± 0.12
F8	3.84 ± 0.14	2.46 ± 0.15	4.72 ± 0.13	3.38 ± 0.11	2.35 ± 0.15

CB – coliforms bacteria, E – enterococci, TCB – total count of bacteria, LAB – lactic acid bacteria, MFF – microscopic filamentous fungi, SD – standard deviation.

A total of 3,758 isolates from cheese “Bryndza” were identified using MALDI-TOF Biotyper (Table 2). The most abundant microbial species isolated from cheese were *Yarrowia lipolitica* (254 isolates), *Lactobacillus paracasei* subsp. *paracasei* (252 isolates), and *Dipodascus geotrichum* (*Geotrichum candidum*, 227 isolates). The most abundant family of cheese “Bryndza” was Lactobacillaceae (25.24%).

**Table 2:** Number of isolated species from F1 to F8

Species	F1	F2	F3	F4	F5	F6	F7	F8	Total
*Acinetobacter baumannii*	10	14	15	18	12	11	9	13	**102**
*Acinetobacter tandoii*	15	18	10	16	15	14	11	10	**109**
*Aspergillus fumigatus*	12	15	14	18	10	12	16	13	**110**
*Bacillus pumilus*	8	9	7	8	6	8	7	9	**62**
*Candida catenulata*	4	6	5	8	9	11	12	8	**63**
*Candida krusei*	6	8	5	7	4	8	9	5	**52**
*Candida lusitaniae*	15	15	16	11	15	17	10	15	**114**
*Candida rugosa*	6	8	9	9	9	7	10	11	**69**
*Candida utilis*	8	5	8	5	9	6	8	10	**59**
*Citrobacter braakii*	8	6	6	8	7	8	5	5	**53**
*Citrobacter koseri*	5	6	8	9	5	4	5	6	**48**
*Dipodascus geotrichum*	25	30	28	32	26	28	30	28	**227**
*Dipodascus silvicola*	6	5	7	4	5	4	3	5	**39**
*Enterobacter cloacae*	2	4	8	9	5	6	4	10	**48**
*Enterobacter ludwigii*	8	9	7	5	6	8	9	5	**57**
*Enterococcus faecalis*	6	11	5	8	9	9	7	8	**63**
*Enterococcus faecium*	10	15	25	24	21	15	12	18	**140**
*Enterococcus hirae*	15	6	18	7	9	10	14	11	**90**
*Escherichia coli*	14	10	12	8	10	12	16	10	**92**
*Hafnia alvei*	5	6	8	7	8	9	10	8	**61**
*Klebsiella oxytoca*	10	5	6	8	9	7	6	8	**59**
*Klebsiella pneumoniae* ssp. *ozaenae*	6	4	8	7	8	8	5	6	**52**
*Klebsiella pneumoniae* ssp. *pneumoniae*	5	6	7	8	7	8	9	5	**55**
*Lactobacillus brevis*	12	15	18	21	16	18	21	18	**139**
*Lactobacillus harbinensis*	8	9	10	8	9	15	12	16	**87**
*Lactobacillus johnsonii*	5	8	8	6	12	4	11	8	**62**
*Lactobacillus plantarum*	23	21	25	18	26	22	20	32	**187**
*Lactobacillus paracasei* ssp. *paracasei*	28	31	39	45	36	15	28	30	**252**
*Lactobacillus paraplantarum*	8	15	15	16	15	17	14	10	**110**
*Lactobacillus suebicus*	5	4	6	5	8	7	9	6	**50**
*Lactococcus lactis* ssp*. lactis*	18	22	25	19	16	8	15	21	**144**
*Lactococcus lactis*	15	10	10	12	14	10	18	14	**103**
*Microbacterium liquefaciens*	8	9	8	9	11	6	4	5	**60**
*Mucor circinelloides*	8	4	6	10	9	8	7	9	**61**
*Pediococcus acidilactici*	6	4	8	11	10	5	8	9	**61**
*Penicillium* sp.	7	5	8	8	6	4	5	8	**51**
*Pichia cactophila*	4	5	6	6	7	8	5	6	**47**
*Raoultella ornithinolytica*	5	7	8	9	10	12	8	5	**64**
*Rhizopus* sp.	8	7	5	4	2	4	6	5	**41**
*Serratia liquefaciens*	6	7	5	8	5	4	6	5	**46**
*Staphylococcus aureus* ssp. *aureus*	6	2	5	4	5	3	4	6	**35**
*Staphylococcus pasteuri*	2	4	6	7	5	4	6	3	**37**
*Stenotrophomonas maltophilia*	5	4	6	5	8	6	4	5	**43**
*Yarrowia lipolytica*	25	28	30	28	39	45	28	31	**254**
**Total**	421	442	499	503	493	455	466	479	**3,758**

**Figure 1:**
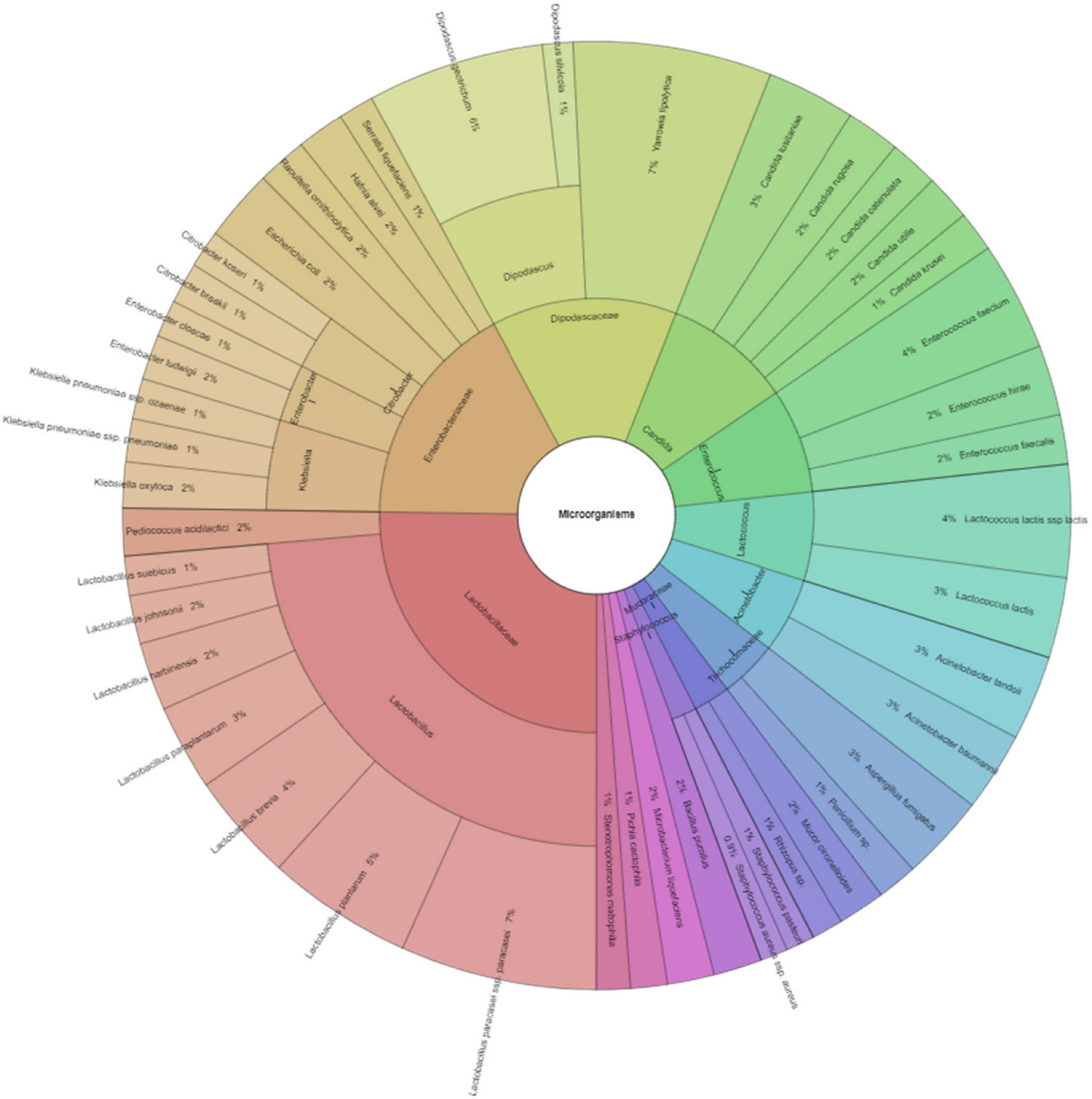
Diversity microorganisms isolated from ewes’ cheese “Bryndza” (outermost ring: species, middle ring: genus, innermost ring: family).

**Figure 2:**
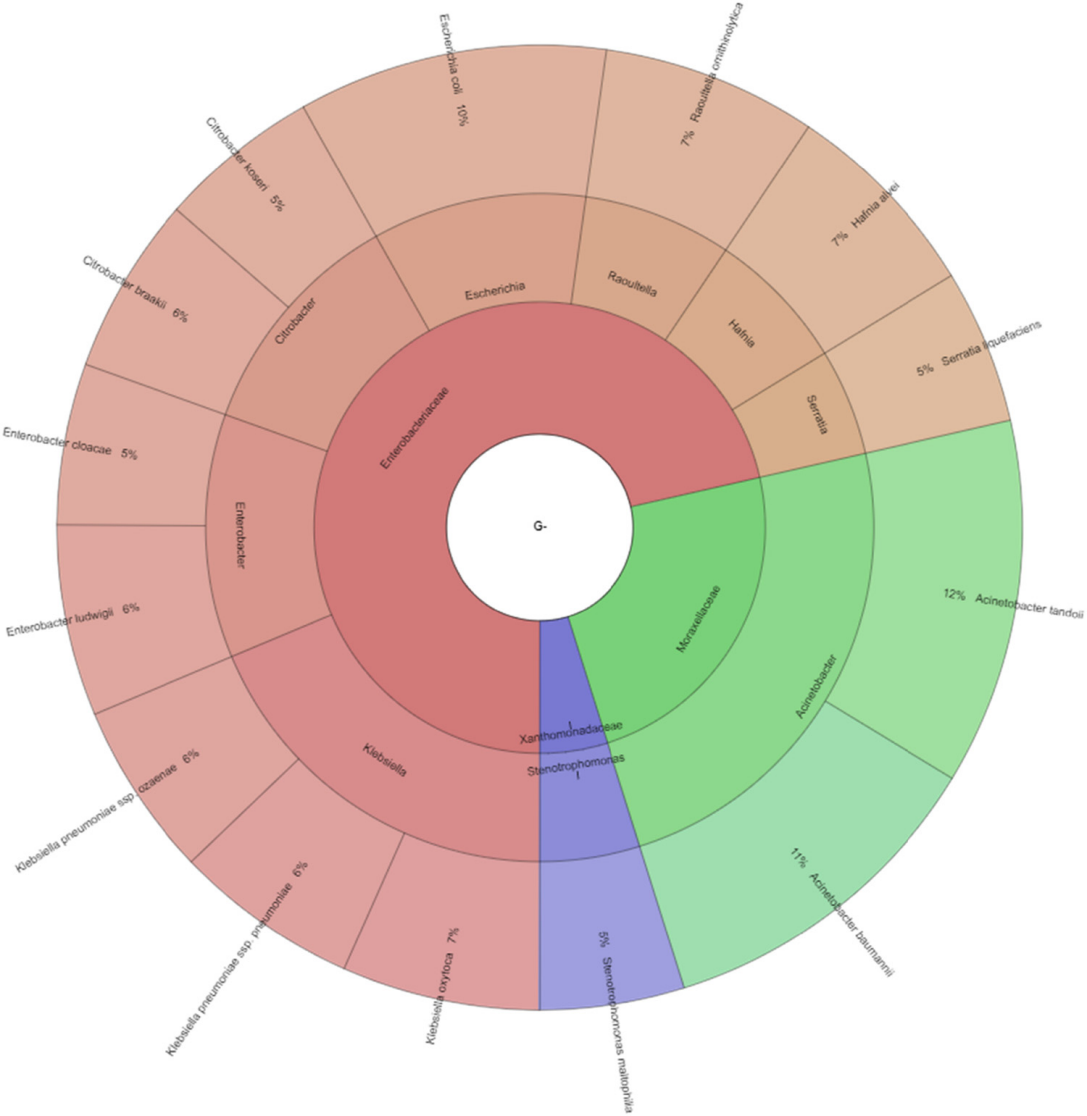
Diversity of Gram-negative bacteria isolated from ewes’ cheese “Bryndza” (outermost ring: species, middle ring: genus, innermost ring: family).

**Figure 3:**
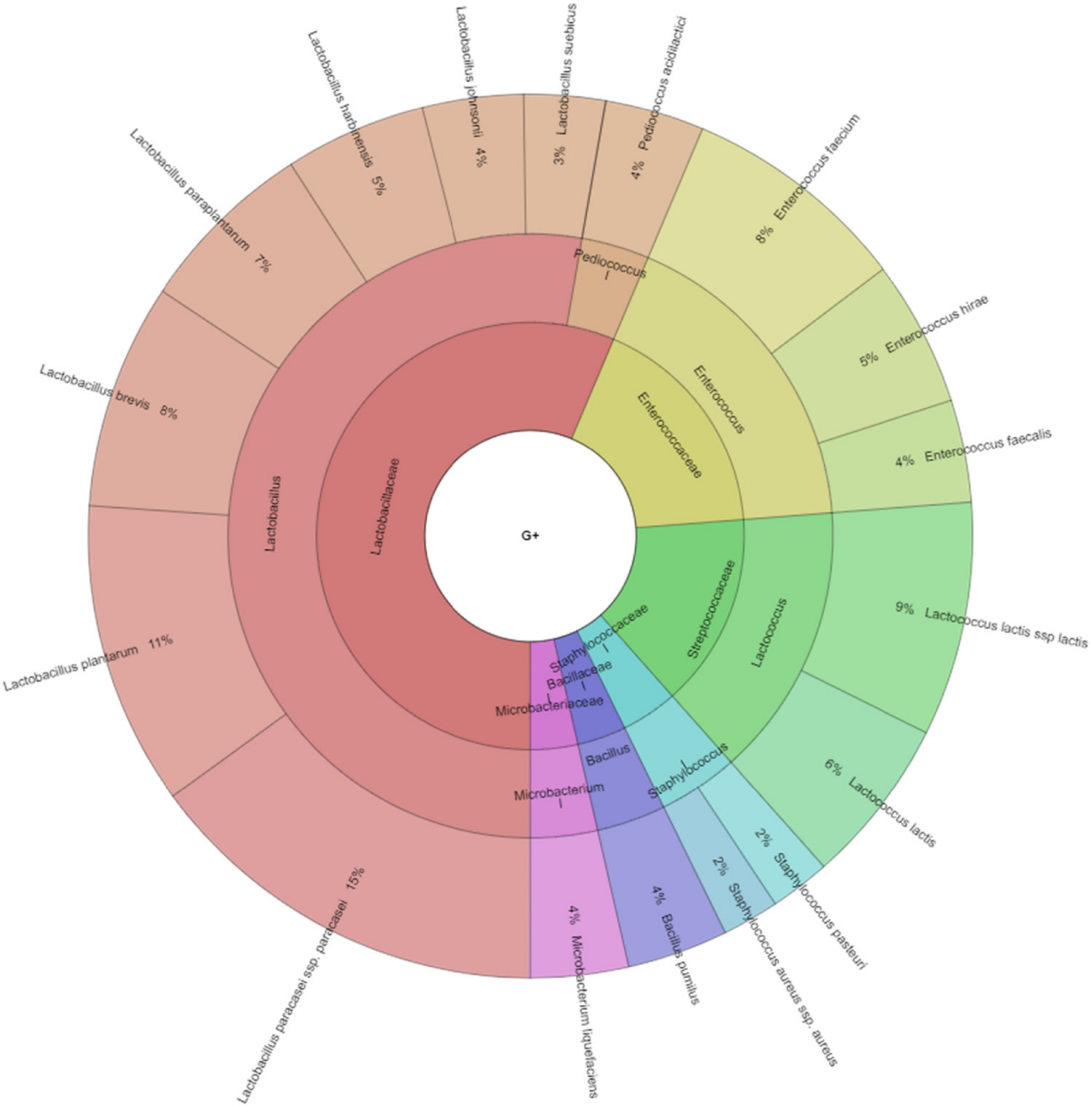
Diversity of Gram-positive bacteria isolated from ewes’ cheese “Bryndza” (outermost ring: species, middle ring: genus, innermost ring: family).

**Figure 4:**
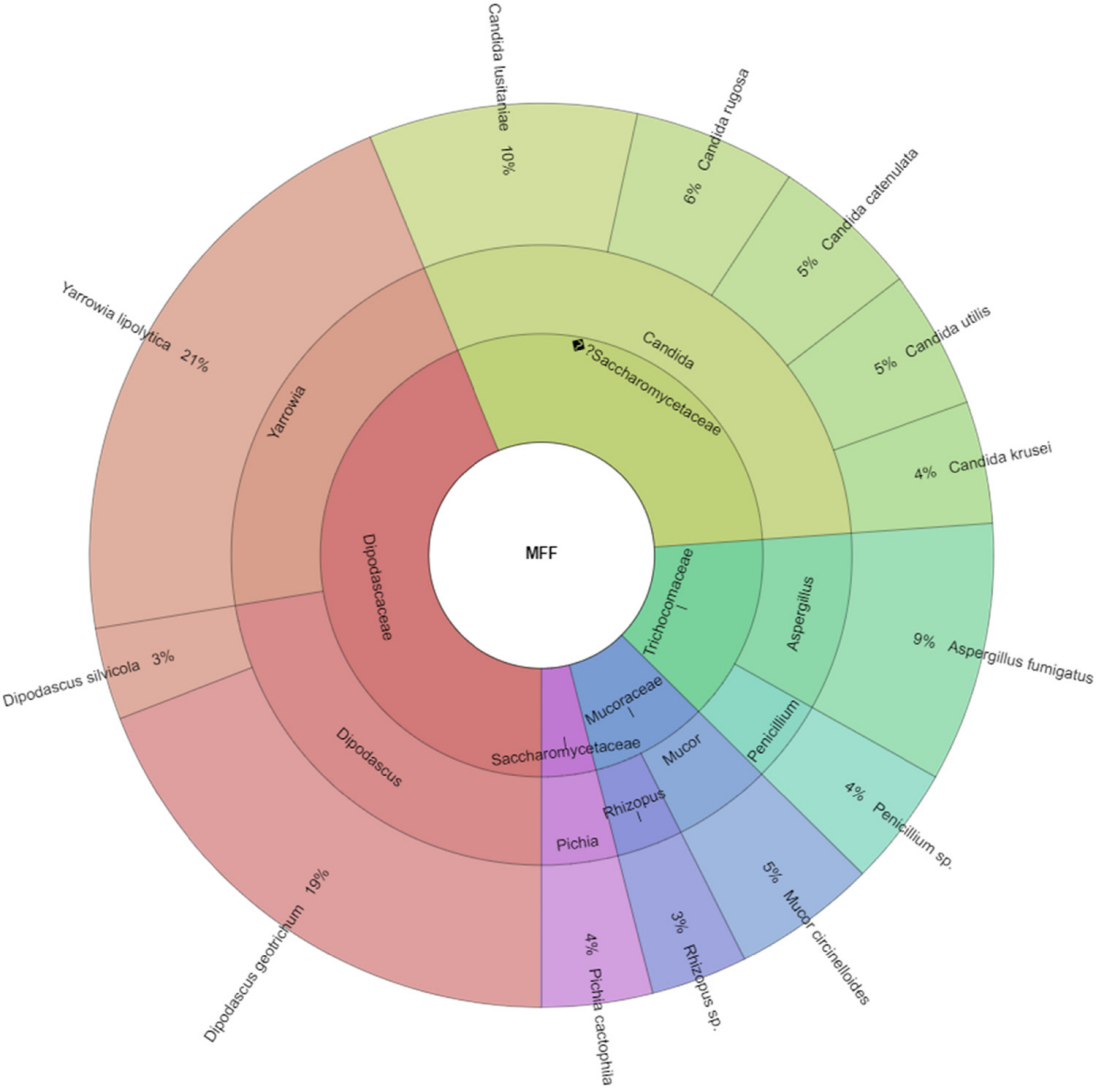
Krona chart for microscopic filamentous fungi isolated from ewes’ cheese “Bryndza” (outermost ring: species, middle ring: genus, innermost ring: family).

In our study, Gram-negative, Gram-positive bacteria, and microscopic filamentous fungi accounted for majority of the identified microbiota in “Bryndza.” The Enterobacteriaceae family was the most abundant family of Gram-negative bacteria isolated. Percentage of all isolated family, genera, and species indicates Krona (Figure 1). Altogether 889 isolates belonged to Gram-negative bacteria group. The Lactobacillaceae family was the most abundant among Gram-positive bacteria (Figure 2). Altogether 1,682 isolates of Gram-positive bacteria were identified. *Yarrowia lipolitica* was the most abundant microscopic fungi (21%) (Figures 3 and 4).

## 4 Discussion

The total count of bacteria in ewes’ cheese “Bryndza” samples was from 4.38 ± 0.15 log cfu/g for F1 to 4.81 ± 0.12 log cfu/g for F6 total count of bacteria. The microbial counts similar to detected here were described previously for ewes’ cheese “Bryndza,” where number of total count of bacteria ranged from 3.87 to 4.32 log cfu/g [10]. Our findings on coliform bacteria counts (3.84 log cfu/g) were in line with Pangallo et al. [13] who reported their counts in the spring “Bryndza” at 3.87 log cfu/g. The counts of coliform and *Staphylococcus* spp. in Šaková et al.’s [3] study reached 10^5^ to 10^6^ cfu/g in “Bryndza” cheese; moreover, the majority of *Staphylococcus* spp. isolates were coagulase-positive (10^4^ cfu/g). Within this study, the coliforms counts were less than 10^5^ cfu/g. Coliforms were frequently identified in dairy products and contamination of dairy products may occur during milk storage, transportation, and processing, if the hygienic requirements are not met [14,15,16,17]. Intrinsic and extrinsic factors such as low pH, salt concentration, and water activity all affect the survival of coliforms and a decline in coliforms counts was detected during the ripening of other cheese types [18,19,20]. *Escherichia coli, Klebsiella* sp., and *Enterobacter* sp. were isolated from local Nigerian soft and semisoft cheese [21], whereas *Citrobacter braakii, Enterobacter sakazakii,* and *E. coli* were identified in the local Italian cheese [22,23]. High microbial counts of 9.0 log cfu/g were detected in raw ewes milk after 30 days of maturation [24]. The most important bacteria for ripening of ewes’ cheese are lactic acid bacteria and several these bacteria in “Bryndza” were considered as potentially probiotic in character [25]. Lactic acid bacteria up to 10.95 log cfu/g in “Bryndza” was reported previously [13] in comparison to 3.62 log cfu/g identified in our study. The microbiological quality of dairy products can be improved by adding a starter culture such as lactic acid bacteria, which will prevent the growth of pathogenic microorganisms [26].

Yeasts counts (2.61 log cfu/g) and *Enterococcus* counts (2.64 log cfu/g) in our study were lower than 5.97 log cfu/g and 7–8 log cfu/g, respectively, detected in other studies [13,14]. Yeasts belong to the natural microbiota of “Bryndza” cheese and contribute to the ripening of the cheese. The differences in yeasts counts in “Bryndza” cheese from raw and pasteurized milk were not significant. *Dipodascus geotrichum* was the predominant yeast identified in the present study in “Bryndza cheese.” The functional significance of this yeast is related to a breakdown of sugars, milk fat, and proteins [27,28]. Some strains of *Dipodascus geotrichum* were reported to form esters and sulfur compounds, important for the development of typical aroma and other characteristics of cheese [29].

*Candida catenulata, C. krusei, C. lusitaniae, C. rugosa, C. utilis, Dipodascus geotrichum, D. silvicola, Pichia cactophila,* and *Yarrowia lipolitica* were isolated in the present study. *Galactomyces/Geotrichum* (*Dipodascus geotrichum*) was reported in “Bryndza” of Slovak origin [30]. Yeasts as compounds of the milk microbiota are important in the agri-food industry as they are involved in biodegradation and depollution, may act as contaminants. *Dipodascus geotrichum* was found as a commensal of gut of humans and animals [31] and is important for milk production since it is naturally present in raw milk [32,33,34,35].

A total of 3758 isolates were identified to species level in cheese “Bryndza” and the most frequently isolated species were *Yarrowia lipolitica*, *Lactobacillus paracasei* subsp. *paracasei*, and *Dipodascus geotrichum* (*Geotrichum candidum*). Lactobacillaceae (25.24%) was the most frequently isolated family of the cheese “Bryndza.” Among the isolates, 889 were Gram-negative, 1,682 were Gram-positive, and 1,187 were microscopic filamentous fungi. In other study [10], a total of 1,175 isolates were identified by mass spectrometry and included Gram-negative bacteria, Gram-positive bacteria, and microscopic filamentous fungi. In the same study [10], 199 isolates were isolated and identified from Gram-negative, 599 isolates from Gram-positive, and 377 isolates of yeast and molds.

Beside the abundance of Lactobacillaceae (25.24%), other isolated families were Bacillaceae (1.65%), Dipodascaceae (13.84%), Enterobacteriaceae (16.89%), Enterococaceae (7.8%), Microbacteriaceae (1.6%), Moraxellaceae (5.61%), Mucoraceae (2.71%), ‎Saccharomycetaceae (10.75%), Staphylococcaceae (1.91%), Streptococaceae (6.57%), Trichocomaceae (4.29%), and Xanthomonadaceae (1.14%). Similar results of identified genera were found [10].

In our study, 44 microbial species representing 24 genera were identified using MALDI-TOF MS Biotyper with score higher than 2.00. *Lactobacillus paracasei* subsp. *paracasei* was identified as most often identified species in the present study. *Lactococcus, Lactobacillus, Streptococcus, Leuconostoc*, and *Enterococcus* were members of lactic acid bacteria group commonly found in cheeses [36]. Needs for characterization of complex population and discovery of new lactic acid bacteria strains facilitate the research on the microbiota present in raw milk cheese and other traditional dairy products [37]. The genus *Enterococcus* was frequently isolated from cheese and identified as the prevalent among the lactic acid bacteria isolated from Coalho cheese in Brazil [38]. Several species of *Enterococcus* genus such as *E. faecium, E. faecalis, E. italicus*, *E. durans*, *E. casseliflavus*, and *E. gallinarum* were isolated from raw milk and cheese [38,39]. The dominant group of bacteria in “Bryndza” was lactic acid bacteria, mainly *Lactobacillus* species [10]. *Lactococcus, Pediococcus, Enterococcus,* and *Streptococcus* were abundant in “Bryndza” from different Slovak regions [3,40,41]. Previous study [42] identified *Klebsiella* (65%), *Escherichia* (20%), *Serratia* (10%), and *Enterobacter* (5%) in cheese samples. *Klebsiella oxytoca, K. pneumoniae* and *K. ornithinolytica,* and *E. coli* were identified among Enterobacteriaceae.

*Aspergilus fumigatus, Mucor circeneloides, Rhizopus* sp., and *Penicillium* sp. from micromycetes were isolated in “Bryndza” in our study. “Bryndza” could serve as a natural habitat for microscopic fungi, but their role in cheese ripening needs to be studied. *Mucor* spp. has been occasionally isolated from sheep cheese, accompanied by recognized human and plant pathogens [43,44,45,46]. Various applications of *M. circinelloides* were reported previously: starter cultures in China and Vietnam, and a contributor to organoleptic proprieties in study in Poland [47,48]. *Mucor* strains are accumulated in lipid bodies rich in polyunsaturated fatty acids [49].

In contrast to industrial food products, traditional cheese is of particular interest to consumers who care about the nature, origin, and nutritional value of foods [50]. Much of their reputation is attributed to the unique organoleptic properties and to the indigenous microorganisms living in raw milk or natural starters [51]. However, raw milk can also contain pathogenic microorganisms that have been raising public health concerns since the beginnings of the dairy industry [27].

## 5 Conclusion

The microbiota of traditional ewes’ cheese produced in Slovakia include diverse families of Bacillaceae, Dipodascaceae, Enterobacteriaceae, Enterococaceae, Lactobacillaceae, Microbacteriaceae, Moraxellaceae, Mucoraceae, ‎Saccharomycetaceae, Staphylococcaceae, Streptococaceae, Trichocomaceae, and Xanthomonadaceae. The most abundant microbial species were *Yarrowia lipolitica, Lactobacillus paracasei* subsp. *paracasei*, and *Dipodascus geotrichum*. The unique sensory properties of the Slovak “Bryndza” are believed to be attributed to microbial activity of the microbiota primary present in the lump ewes’ cheese – *Lactobacillus* spp., *Lactococcus* spp., *Streptococcus* spp., *Enterococcus* spp., *Kluyveromyces marxianus,* and *Dipodascus geotrichum*. The results of this study contribute to better understanding of the microbiota of the local cheese produced in Slovakia. The study of “Bryndza” microbiota is important for development of processing technology and improvements in safety of products.
